# The ethics of open access publishing

**DOI:** 10.1186/1472-6939-14-16

**Published:** 2013-03-22

**Authors:** Michael Parker

**Affiliations:** 1 The Ethox Centre, Department of Public Health, University of Oxford, Old Road Campus, Oxford, OX3 7LF, United Kingdom

**Keywords:** Ethics, Bioethics open access, Publishing ethics, Gold model, Universities humanities

## Abstract

Should those who work on ethics welcome or resist moves to open access publishing? This paper analyses arguments in favour and against the increasing requirement for open access publishing and considers their implications for bioethics research. In the context of biomedical science, major funders are increasingly mandating open access as a condition of funding and such moves are also common in other disciplines. Whilst there has been some debate about the implications of open-access for the social sciences and humanities, there has been little if any discussion about the implications of open access for ethics. This is surprising given both the central role of public reason and critique in ethics and the fact that many of the arguments made for and against open access have been couched in moral terms. In what follows I argue that those who work in ethics have a strong interest in supporting moves towards more open publishing approaches which have the potential both to inform and promote richer and more diverse forms of public deliberation and to be enriched by them. The importance of public deliberation in practical and applied ethics suggests that ethicists have a particular interest in the promotion of diverse and experimental forms of publication and debate and in supporting new, more creative and more participatory approaches to publication.

## Main text

Scholarly journals have been at the heart of academic life since the publication of the *Journal des sçavans* and the *Philosophical Transactions of the Royal Society* in the middle of the 17^th^ Century. The centrality of deliberation, critique and contestation to academic knowledge production means that publication will always be an important part of academic work. The form taken by such publishing, its economic basis, and its relation to the processes of knowledge production and consumption are, however, currently very much in question as the subscription-based journal increasingly comes under threat from moves towards open-access models of publishing. In the context of biomedical science major funders such as the Wellcome Trust and the US National Institutes of Health are mandating open access as a condition of funding and such moves are also increasingly common in other disciplines [1]. Recently, for example, the UK Government-convened Working Group on Expanding Access to Published Research Findings - the ‘Finch Committee’ - has argued that open access should be adopted across the full range of UK research [2]. Whilst there has been some debate about the implications of open-access for the social sciences and humanities, there has been little if any discussion about the implications of open access for ethics. This is perhaps surprising given both the central role of public reason and critique in ethics and the fact that many of the arguments made for and against open access have themselves been couched in moral terms. In what follows, I explore arguments in favour and against moves towards open access publishing and illustrate the relevance of these arguments to ethics research.

### Arguments for open access publishing

By far the most common argument made for open-access by scientists and science funders is one that springs from a widely-held belief that the sharing of data will lead to more rapid scientific progress and a reduction in unnecessary duplication of scientific effort. In the context of the humanities and social sciences these arguments are sometimes complemented by those emphasising the important role of open and critically reflective communities of practice and the potential for open access to support the creation of spaces for constructive criticism and epistemological economies of scale which mean that research can be deeper, richer, broader, and – where appropriate – faster. Whilst greater speed may not always, or even often, be conducive to better public reason on moral matters, it is nonetheless sometimes important for ethical deliberation to be timely – for example in the case of the proposed introduction of novel biotechnologies or changes in Government policy. Furthermore, the role of ethics in critically reflecting upon all of the normative arguments pertinent to important moral questions suggests that ethicists and those concerned with public reason in ethics ought to be strongly in favour of the availability of both empirical and normative literature relevant to the question under consideration. To the extent that access to important arguments is limited public reason is necessarily in this regard impoverished.

A second argument in favour of open-access, particularly – but not only - in the context of publically funded research, is one grounded in claims about obligations of reciprocity. The findings of research funded by the public should, it is argued, be made freely available to those who have paid for it. This is an argument about the nature of the researcher’s social licence to practice and the responsibilities of academics and those who spend public money to contribute to the dissemination of knowledge. The current subscription based model of scholarly publication limits access to those in institutions or with the resources to cover the cost of subscriptions. Open access, by contrast, reflects the social value of increased public understanding of, engagement with, and involvement in academic knowledge production. The argument from reciprocity is of course particularly relevant in the context of research, including that in ethics, carried out in low-income settings. Where research is carried out in such settings there is for example an obligation to ensure as far as is possible that resulting publications are available to colleagues in low-income settings, and where appropriate to relevant policy makers. This suggests that such research should wherever possible be published in on-line open access journals. In the context of medical research, there is an increasing emphasis on the ethical requirement for such research to have ‘social value’ [3]. There seems no good reason why this ought not to be equally the case in ethics.

Third, it has been argued that subscription-based models of publishing are unfair and exploitative because they require publically-funded academics to submit their work to commercially run journals, to carry out peer-review and sit as members of editorial boards, and then to pay to access the fruits of their own intellectual labour. The monopolistic position of publishers of academic journals means, it is argued, that they profit unfairly out of the - free - labour of academic researchers because they are able to place restrictions on who can gain access to knowledge and who is to count as a knowledge producer.

Finally, and relatedly, some have suggested that the current subscription-based system is inherently conservative and has the potential to lead to a narrowing of intellectual and scholarly life. By contrast, models of open-access publishing offer the possibility of moving away from an approach to publication driven by the branding and marketing imperatives of journals and their overly rigid views about disciplinary boundaries and publication formats. Open access initiatives have the potential to lead to the development of new, creative and more productive communities of publishing practice and the generation of new forms of understanding and research collaboration. This argument, coupled with the first argument above – about the potential for open access to enrich moral debate – is likely to be particularly attractive to those working in ethics. For, whilst, philosophically minded ethicists are rightly wary of arguments placing special value on majority views, it is nonetheless important for the quality and relevance of moral deliberation that as wide a range as possible of relevant arguments and claims is brought into the domain of public reason. Open access initiatives in ethics have the potential to ensure that debate is enriched by a far wider range of potentially relevant arguments.

### Arguments against open access publishing

The claim that open access will encourage more and better research is an *empirical* claim. But, will open access in fact generate more, better, faster research? While there may be good reasons for believing that it will, this should not be taken for granted. In the context of biomedical research, for example, the potential for open-access to fail to support sustainable scientific collaboration is explicitly acknowledged in the Fort Lauderdale Agreement (one of the founding open-access manifestos in biomedical science) which highlights the crucial role of ‘scientific etiquette’ in sustainable scientific collaboration [4,5]. Such considerations need to be taken seriously. At the very least, it is argued, there is a need to develop an evidence-base around which models of publishing are most likely to lead to sustainable high quality research and this cannot be achieved effectively in the context of a blanket imposition of a universal open access model. Such considerations about the compatibility of open access with sustainable research collaboration are also clearly of important to those working in ethics. Whilst this is not, in the context of ethics, generally going to be a question of the potential for the rapidity of open-access publishing and data release to undermine emerging capacity, as it is for the scientist, equity and the sustainability of collaboration are hugely important in ethics. For example, it is important that those who contribute to collaborative ethics research are appropriately acknowledged, that decisions about authorship are made fairly, and that researchers who are less well-resourced are not further disadvantaged by their participation in collaborative research or open access publishing. Open access should for example be genuinely open and accessible to those working in low-income settings. To take one example, are open access publications currently available in a mobile device readable format?

Many of the arguments against open access have been targeted at what has come to be known as the ‘Gold Model’ – a model which has already been adopted by some journals and was recently favoured by the United Kingdom’s Finch Committee. In brief, the Gold Model involves a shift from the current subscription-based approach to one in which authors (which in practice means their institutions) pay to cover the costs of (open access) publishing. One argument that has been made against this model is that like the subscription model it may in some cases favour the financial interests of publishers. This, it is argued, is a model in which the public continues to pay both for the production of knowledge and for access to it and from which commercial publishers will continue to profit because the costs charged by the journals do not reflect the true costs as much of the key labour will continue to be carried out for free by publically funded academics. Whilst this may in itself be insufficient reason to reject the Gold Model, it does suggest that if it is to be justified it will need to be complemented by transparency about its real costs to ensure good value for public money. A further, related argument against a formal requirement for publically-funded researchers and research institutions to publish in open access journals is that this will mean that the private sector will have unlimited access to publically funded research but no reciprocal responsibility to make its own findings, which may have been made possible by access to the fruits of publically funded research, freely available. Were this asymmetry to persist, private research organisations and commercial publishers would be able to benefit unfairly from a publically funded knowledge subsidy. Clearly such arguments would have less force were there to be a shift towards the view that privately funded research, particularly that which benefits through access to publically funded research ought itself to be made available in open access formats.

Another argument that has been made against the Gold Model of open access is that it has the potential to lead to the emergence of a conservative approach to publishing driven by the needs of a small number of high impact journals and by the requirements of forms of academic audit such as the UK’s Research Excellence Framework. Against this background, the Gold Model of open access might have only very limited potential to generate the increased discussion and better and faster knowledge production promised by its advocates and might contribute still further to the narrowing of debate and of the forms of academic knowledge production: limiting the freedom of open access to be truly innovative and open. A related claim is that the author pays approach epitomised by the Gold Model would further entrench divisions between richer and poor institutions and would further institutionalise knowledge production by widening the divide between independent researchers and those affiliated with academic institutions. They will be separated by the costs of publication rather than by those of subscription. Paradoxically, just as more data are being made publically available by open access models of data-release it may become less easy for independent researchers to publish the results of their research using these data. Whilst it is clear that the ethos of many existing open-access journals, such as for example BMC Medical Ethics, is informed by a commitment to the promotion of broad and inclusive debate, such a possibility needs nonetheless to be taken seriously. One way in which these concerns might perhaps be alleviated is though the wider use of fee waivers or through the availability of means-tested subsidies.

## Conclusion: what, if anything, is special about ethics?

Should those who work on ethics welcome or resist moves to open access publishing? Whilst all of the arguments above are relevant to ethics, it seems undeniable that those who are working in areas of practical and applied ethics – such as for example, bioethics – ought to place particular value on the potential for their research to contribute to, and to benefit from, public deliberation. Whilst all academic knowledge has the potential to be of public value, applied ethics is specifically concerned with the normative analysis of issues relevant to contemporary policy and practice and with the promotion of public discussion. This would suggest that, whatever their views about particular models of open access, those who work in ethics ought to have a strong interest in supporting moves away from subscription-based publishing (particularly when the price of such subscriptions is well beyond the reach of most members of the public) towards more open approaches which have the potential both to inform and promote rich and diverse forms of public deliberation and to be enriched by them. Whichever model ends up being finally adopted in the wider academy, the importance of public deliberation in practical and applied ethics suggests that ethicists ought also to have a particular interest in the promotion of diverse and experimental forms of publication and debate and in supporting new, more creative and more participatory approaches to open access publication that reach beyond any that are likely to be commercially viable or profitable.

## Competing interests

I declare that I have no competing interests.

## Pre-publication history

The pre-publication history for this paper can be accessed here:

http://www.biomedcentral.com/1472-6939/14/16/prepub
